# Modulation of Altered Immune Parameters IL-2 and TNF-α in Diabetic Animal Models: A Therapeutic Insinuation of Metformin Beyond Diabetes

**DOI:** 10.7759/cureus.45216

**Published:** 2023-09-14

**Authors:** Akhtar Ali, Shehla Shaheen, Muhammad Z Imran, Zahida Memon, Nisha Zahid, Farah Ahmad, Abdul Hameed

**Affiliations:** 1 Pharmacology, Ziauddin Medical College, Ziauddin University, Karachi, PAK; 2 Hematology, Ziauddin University Hospital, Ziauddin University, Karachi, PAK; 3 Pharmacology and Toxicology, Sapienza University of Rome, Rome, ITA; 4 Community Health Sciences, Ziauddin University, Karachi, PAK; 5 Molecular Medicine, Ziauddin Medical College, Ziauddin University, Karachi, PAK

**Keywords:** animal model, healthy, diabetic, metformin, immunomodulation

## Abstract

Background: Immunomodulatory drugs target the overall immune system, hence producing numerous toxic effects on the other organs with serious health manifestations. Due to these safety concerns, there is a need to introduce or repurpose a new drug with immunomodulatory effects with good safety, efficacy, and better tolerance. Metformin, a standard antidiabetic drug, was evaluated for its immunomodulatory effects in diabetic models in the current study.

Methodology: The diabetic model was developed by intraperitoneal (IP) administration of streptozotocin (60 mg/kg). The experimental rats were divided into six groups (three diabetic and three non-diabetic) with six rats in each group. Metformin (50 mg/kg and 80 mg/kg) was given orally to both diabetic and non-diabetic groups, once a day, for 42 days. Immunomodulatory cytokines interleukin (IL)-2, IL-4, IL-5, tumor necrosis factor (TNF)-α, and interferon gamma (INF-ɣ) were analyzed from blood samples by BD FCAP flow cytometer.

Results: The results revealed a significant (p=0.002) decrease in IL-2 and TNF-α in diabetic groups in comparison to control rats. However, no significant changes were observed in IL-4, IL-5, and INF-ɣ levels. Importantly, the treatment of metformin at both doses, i.e., 50 and 80 mg/kg, significantly reduced the elevated levels of IL-2 and TNF-α when compared to untreated diabetic groups.

Conclusion: Metformin may be considered as an optimum drug candidate to reduce pro-inflammatory cytokines, IL-2 and TNF-α, that can lead to the reduction of long-term diabetic complications.

## Introduction

In 1950, Jean Sterne, a French scientist, published about the properties of metformin, a “biguanide.” Due to its glucose-lowering property, he named it “glucophage.” Considering its lower side effect profile, weight-lowering properties and extended-release formulation metformin became the first-choice drug for diabetes at the dose range of 500-2,500 mg/day [1,2]. The proposed mode of action of metformin in the control of blood glucose includes a decrease in hepatic glucose production and its intestinal absorption. Simultaneously, it also increases insulin sensitivity by increasing peripheral glucose uptake and utilization without causing hypoglycemia, which is a major side effect of other oral hypoglycemic agents. Metformin suppresses adenosine triphosphate (ATP) production by inhibiting complex I of the respiratory chain in mitochondria and mediates its actions via both adenosine monophosphate (AMP)-activated protein kinase (AMPK)-dependent and AMP kinase-independent pathways at the cellular level [3].

The uses and clinical implementations of metformin are being studied for different disorders such as polycystic ovarian syndrome (PCOS) and myocardial infarction, and additionally, in 2001, its beneficial effects were documented in ovarian, breast, prostate, and colorectal cancers. The anticancer and antiaging effects of metformin are attributed to its ability to activate the AMP kinase enzyme system [4]. It was reported in 2014 that metformin suppresses tumor progression due to its activity on the AMP kinase enzyme system, and it was found that it can alter the levels of immune cells, interleukins (ILs), and cytokines such as CD8+, interleukin-2 (IL-2), tissue necrosis factor-α (TNF-α), and interferon gamma (INF-ɣ) [5]. Another study carried out in 2017 on breast cancer showed that the anticancer effects of metformin are due to its ability to enhance IL-2, IL-4, and IL-10 activity [6]. In these studies, it was proposed that the possible mechanism behind the anticancer effect of metformin is due to its immune-modulating properties [7]. Furthermore, in a clinical trial conducted on diabetic patients with and without tuberculosis infection, it was identified that metformin increased the activity of CD8+ cells, INF-ɣ, and TNF-α, and it was also highlighted that the effects of metformin may vary depending on the dose as well as the duration of treatment [8].

Immune disorders including both immune deficiency and autoimmune diseases such as systemic lupus erythematosus (SLE), multiple sclerosis, rheumatoid arthritis, and acquired immunodeficiency syndrome (AIDS) need treatment with immunomodulatory drugs for longer periods. These drugs target the overall immune system and hence produce numerous toxic effects and are also quite expensive [9]. Although metformin is the most commonly prescribed oral hypoglycemic drug, the majority of its effects are attributed to its glucose-lowering properties. Its direct effects on the immune system have not been documented yet, and as reported in the management of various cancers, it modulates the immune parameters. Considering its actions on the immune system, it is pertinent to evaluate the effects of metformin on various immune cytokines to explore its role as a potential immunomodulatory drug.

## Materials and methods

Materials

Metformin and streptozotocin were obtained from Sigma (St. Louis, MO, USA). BD CBA cytokine kit Th1/Th2 (catalog number: 551827) was used for the analysis of various cytokines, IL-2, IL-4, IL-5, INF-ɣ, and TNF-α.

Animal handling

Male albino rats, aged nine weeks old, weighing about 300-400 g were purchased from Aga Khan Animal Laboratory, Karachi, Pakistan. The animals were given free access to food and water and kept at standard conditions of 20°C-22°C and a 12-hour dark and light cycle. Furthermore, the animals have been handled according to the 2010 Committee on Animal Research and Ethics (CARE) guidelines for handling animals in experiments [10]. Ethical approval was taken from the Animal Ethics Committee of Ziauddin University, Karachi, Pakistan, and protocol number 2018-003 was issued by the committee.

Development of the diabetic model

Nicotinamide dissolved in normal saline, 90 mg/kg (9 mg/100 g) body weight, was administered intraperitoneally (IP), followed by administration of 65 mg/kg (6.5 mg/100 g) body weight streptozotocin dissolved in citrate buffer intravenously [11,12]. On the third day of streptozotocin administration, fasting blood glucose levels were checked and compared with the controls to confirm the accuracy of the procedure. After confirmation of the induction of diabetes, the experimental animals were divided into groups.

Group distribution

Rats were divided into six groups with six rats in each group. Among the six, three were healthy models (i.e., groups 1, 2, and 3), and the remaining three were diabetic models (i.e., groups 4, 5, and 6).

Healthy Models

The healthy models were as follows: group 1 (control without intervention), in which only normal saline was administered orally twice a day; group 2, in which metformin (50 mg/kg) dissolved in 5 mL normal saline was administered orally twice a day; and group 3, in which metformin dissolved in 5 mL normal saline was administered at 80 mg/kg orally twice a day.

Diabetic Models

The diabetic models were as follows: group 4 (without intervention), in which only normal saline was administered orally twice a day; group 5, in which metformin dissolved in 5 mL normal saline was administered at 50 mg/kg dose orally twice a day; and group 6, in which metformin 80 mg/kg dose dissolved in 5 mL normal saline was administered orally twice a day.

Treatment methodology

The metformin doses were administered orally through a gavage needle twice a day up to 42 days as follows: 0.5 mL normal saline to groups 1 and 4, 50 mg/kg metformin to groups 2 and 5, and 80 mg/kg metformin to groups 3 and 6 [13]. Following 42 days of intervention, blood samples (2 mL/rats) were collected from the overnight fasted (12 hours) rats as described previously [14]. In order to minimize the pricking pain during the induction of diabetes and sample collection procedures, lidocaine topical gel was used. After the completion of the procedure, euthanasia was performed by intraperitoneal injection of pentobarbital diluted in normal saline [15].

Flow cytometric analysis

The intracellular levels of cytokines (IL-2, IL-4, IL-5, INF-ɣ, and TNF-α) were analyzed from a buffy coat obtained after centrifugation of the blood. The cytokines were analyzed by BD cytometric bead array (CBA) using a mouse Th1/Th2 cytokine kit (catalog number: 551287) by using a BD FACSCalibur flow cytometer. BD FCAP V.01 software was used to investigate the flow cytometric analysis of cytokines. The cytokine levels were calculated according to manufacturer guidelines at 1:4 dilution.

Statistical analysis

For numeric variables, mean and standard error of the mean (SEM) were calculated. The Shapiro-Wilk test was applied to verify the normal distribution of the data. According to this, our data was found to be non-parametric; therefore, the Kruskal-Wallis test was applied to compare the levels of all immune parameters for inter- and intra-group comparisons, followed by post hoc analysis. Data regarding fasting blood glucose and weight analysis was normally distributed. Paired t-test was applied for both of these parameters to analyze pre- and post-interventional effects. A p-value of 0.05 was considered significant at a 95% confidence interval.

## Results

Confirmation of the induction of diabetes for experimental intervention

**Figure 1:**
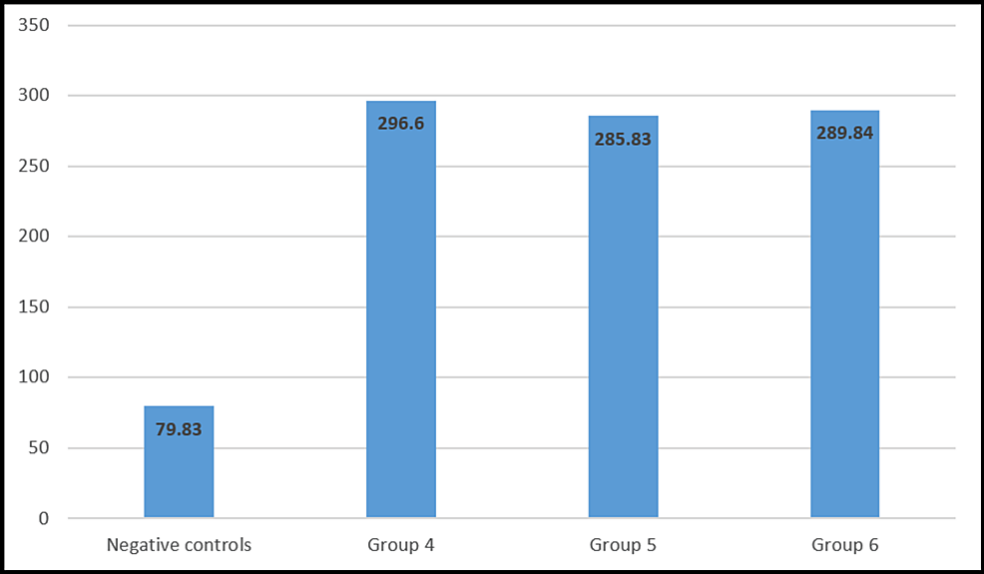
Mean FBS level after streptozotocin administration Figure 1 shows the comparison among FBS levels of negative controls (group 1) with groups 4, 5, and 6 after the administration of streptozotocin. FBS: fasting blood sugar

On the third day of streptozotocin (60 mg/kg) induction, the diabetic rats were selected by comparing their fasting blood glucose with the normal rats. This comparison confirmed the induction of diabetes in groups 4, 5, and 6 with a highly significant (p<0.001) value. Figure 1 demonstrates the mean fasting blood glucose level of the normal control group and diabetic control. These diabetic rats were divided into three groups with six rats/group as follows: untreated diabetic, diabetic treated with metformin at 50 mg/kg, and diabetic treated with metformin at 80 mg/kg.

The effect of metformin on lowering fasting blood glucose in the diabetic model

The pre- and post-interventional analysis of fasting blood glucose levels is demonstrated in Figure 1. A significant increase in fasting blood glucose was observed in untreated diabetic group 4 compared to the untreated non-diabetic group 1.

**Figure 2:**
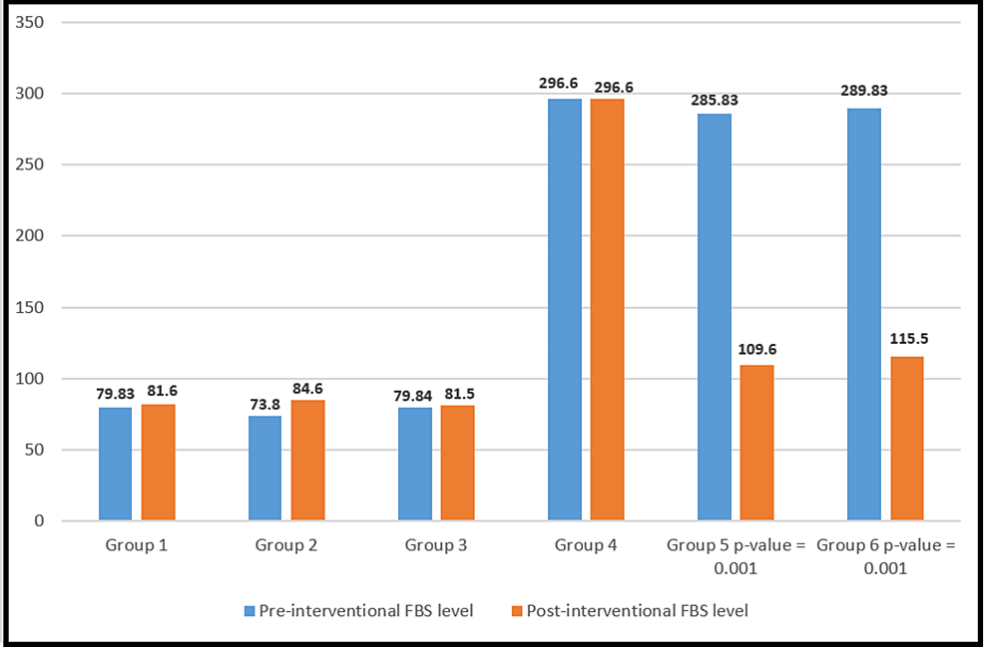
Pre- and post-interventional mean FBS levels Figure 2 shows no effect of metformin on FBS levels in healthy treated groups (groups 2 and 3) when compared to negative controls (group 1), while there was a significant decrease in FBS levels in diabetic treated groups (group 5 and 6) when compared to negative control (group 4). FBS: fasting blood sugar

Interestingly, the fasting blood glucose was significantly (p=0.001) reduced in both metformin-treated groups (group 5 (50 mg/kg) and 6 (80 mg/kg)) when compared to the untreated diabetic control (group 4). However, no change in fasting blood glucose levels was observed in metformin-treated healthy groups (groups 2 and 3) in comparison to the untreated non-diabetic group (group 1). These pre- and post-intervention are represented in Figure 2.

Comparative flow cytometric analysis of cytokines was evaluated following metformin treatment in the blood of treated non-diabetic groups (groups 2 and 3) and untreated non-diabetic group (group 1). The results showed that there was no significant (p>0.05) change in the levels of cytokines (IL-2, IL-4, IL-5, TNF-α, and INF-ɣ) both at 50 mg/kg and 80 mg/kg doses of metformin (Table 1).

**Table 1:** Cytokine levels (pg/mL) and significance among negative control and healthy groups In Table 1, a comparison of cytokine levels in the negative group (group 1) and healthy treated groups (groups 2 and 3) demonstrates a non-significant (p=0.192) increase in IL-2, IL-4, IL-5, TNF-α, and INF-ɣ. This comparison demonstrated that metformin administration in healthy rats did not affect the levels of tested cytokines. IL: interleukin, TNF-α: tumor necrosis factor-α, INF-ɣ: interferon gamma, SEM: standard error of the mean

Cytokines (pg/mL)	Negative controls (group 1) (mean±SEM)	Metformin 50 mg (group 2) (mean±SEM)	Metformin 80 mg (group 3) (mean±SEM)	p-value
IL-2	3210±329	3310±321	3191±344	0.192
IL-4	2291±291	2381±217	2298±281	
IL-5	3729±.412	3819±391	3899±318	
TNF-α	4102±418	3990±352	4231±415	
INF-ɣ	2218±229	2172±261	2196±279	

Furthermore, the IL-2 and TNF-α levels were significantly (p<0.01) increased in untreated diabetic rats (group 4) compared to untreated non-diabetic rats (group 1) (Table 2). However, no significant alteration was observed in IL-4, IL-5, and INF-ɣ (p>0.05). These results suggest that the levels of IL-2 and TNF-α are high in diabetes but without any fluctuation in IL-4, IL-5, and INF-ɣ levels.

**Table 2:** Cytokine levels (pg/mL) in negative and positive control groups Table 2 shows a comparison of cytokine levels in the negative group (group 1) and positive control group (group 4) that highlights a significant (p=0.002) increase in IL-2 and TNF-α levels in the positive control group. This comparison demonstrated that diabetes may lead to an increase in the levels of IL-2 and TNF-α without altering the levels of IL-4, IL-5, and INF-ɣ (p>0.05). IL: interleukin, TNF-α: tumor necrosis factor-α, INF-ɣ: interferon gamma, SEM: standard error of the mean

Cytokines (pg/mL)	Negative controls (group 1) (mean±SEM)	Positive controls (group 4) (mean±SEM)	p-value
IL-2	3210±329	3781±374	0.002
IL-4	2291±291	2381±284	0.481
IL-5	3729±412	3921±213	0.139
TNF-α	4102±418	4729±438	0.002
INF-ɣ	2218±229	2172±182	0.271

Table 3 represents the effect of metformin on immunomodulatory parameters. Interestingly, a significant (p<0.001) reduction in the levels of IL-2 and TNF-α was observed in metformin-treated diabetic groups (groups 5 and 6) in comparison to the untreated diabetic group (group 4). However, IL-4, IL-5, and INF-ɣ levels were not altered at either dose.

**Table 3:** Comparison of positive controls with metformin-treated diabetic groups Table 3 demonstrates the significant decline in the levels of IL-2 and TNF-α (p=0.001) after metformin administration at 50 mg/kg and 80 mg/kg doses in groups 5 and 6. IL: interleukin, TNF-α: tumor necrosis factor-α, INF-ɣ: interferon gamma, SEM: standard error of the mean

Cytokines (pg/mL)	Positive controls (group 4) (mean±SEM)	Metformin 50 mg/kg (group 5) (mean±SEM)	Metformin 80 mg/kg (group 6) (mean±SEM)	p-value
IL-2	3781±374	3381±298	3219±251	0.001
IL-4	2381±284	2488±226	2311±291	0.199
IL-5	3921±213	3858±298	3812±223	1.184
TNF-α	4729±438	4146±336	4271±286	0.001
INF-ɣ	2172±182	2252±195	2279±159	0.371

## Discussion

Recent advancements have reported that behind the development and progression of diabetes, certain immune-mediated mechanisms are involved. Currently, researchers are investigating anti-cytokine agents that can target interleukin pathways involved in inflammation mediated by diabetes. Furthermore, it has been postulated that targeting low-grade inflammation by anti-cytokine drugs can prevent micro- and macrovascular complications of type 2 diabetes [16]. Metformin, being first-line therapy in type 2 diabetes, mediates its antidiabetic effects by 5′ adenosine monophosphate-activated protein kinase (AMPK). Evidence-based studies have documented that AMPK modulates the immune system and inhibits inflammatory pathways by inhibiting cytokine-stimulated nuclear factor κB pathways and downregulating JAK/STAT signaling pathways. AMPK also participates in the modulation of T lymphocytes and other essential cells of the innate immune system [17].

As documented data highlights the role of metformin as an immunomodulatory drug, it is pertinent to assess its effects on different cytokines so that it can be repurposed as an immunomodulatory drug and the financial burden and adverse effects of anti-cytokine drugs on patients with type 2 diabetes can be avoided. To evaluate the effects of metformin as an immunomodulator drug in relation to diabetes, we established a streptozotocin-induced diabetic model because metformin exerts its effects exclusively at stimulatory glucose, but not at basal glucose. Also, pro-inflammatory cytokines are elevated in diabetic conditions [18,19].

In the diabetic model, metformin at both doses of 50 and 80 mg/kg body weight significantly reduced elevated blood glucose (Figure 1); however, no significant changes were observed in non-diabetic healthy model rats (Figure 2). The results of our study and the experimental evidence have already reported that the metformin-induced reduction in glucose is due to its effects on the regulation of liver glucose production, intestinal glucose uptake, and insulin sensitivity [19]. There was no significant effect on glucose levels in the healthy model because of the absence of any considerable role of metformin in direct insulin secretion [20].

A significant increase in IL-2 and TNF-α levels was observed in diabetic rats (group 4) when compared to non-diabetic rats (group 1) (Table 1 and Table 3). Mirroring our data, the reported data by Crook et al. showed that multiple inflammatory pathways are activated during the course of diabetes, leading to persistent low-grade, chronic inflammation and the release of pro-inflammatory cytokines including IL-1, IL-2, IL-6, and TNF-α [21]. Furthermore, the diabetic condition affects normal cellular homeostasis, leading to the depletion of energy and the suppression of immune activity with a subsequent decline in cytokine levels [22]. Hence, the reported data and our preliminary results regarding the elevated levels of IL-2 and TNF-α suggest the role of these key players in serious manifestations in diabetic subjects. An alternative explanation is that IL-2 and TNF-α seem to be the key markers identified by our study for immunomodulation in diabetic subjects.

Based on our experimental evidence of the elevation of IL-2 and TNF-α levels in diabetic subjects, we decided to take metformin one step further to evaluate its effect to overcome this immunomodulation in the type 2 diabetic model. In this respect, we also took the reported data into consideration as well. In prostate cancer, metformin was reported to induce the suppression of pro-inflammatory cytokines IL-2, TNF-α, and INF-ɣ, which was correlated with the inhibition of the mTOR Pathway [23]. In an animal model of multiple sclerosis, the effects of metformin were assessed on Th17 and regulatory T (T-reg) cells, emphasizing that metformin can alter inflammation by interfering with the inflammatory cascade initiated by T-reg and Th17 cells [24]. Calixto et al. investigated the role of metformin in the modulation of cytokines in obese, lean, and control animal models, which states that there is no change in IL-5 and TNF-α levels in the control group, while the increase in these cytokines was recorded in an obese and lean animal model [25].

It has been documented that metformin interferes with immune parameters, and its anticancer effects are attributed to its immunomodulatory activity [26]. Our results revealed no significant difference in the levels of cytokines in untreated non-diabetic rats (group 1) and metformin-treated non-diabetic rats (groups 2 and 3). However, most interestingly, the administration of metformin at both 50 and 80 mg/kg doses significantly (p<0.001) suppressed the alleviated levels of cytokines, i.e., IL-2 and TNF-α, in groups 5 and 6, respectively, when compared to positive controls (group 4) (Table 1). Hence, according to the results of the current study, metformin seemed to be a promising drug to reduce pro-inflammatory cytokines (IL-2 and TNF-α).

## Conclusions

The induction of diabetes mellitus leads to the initiation of the inflammatory process and the release of pro-inflammatory cytokines such as IL-2 and TNF-α and does not produce any change in the levels of IL-4, IL-5, and INF-ɣ. Intervention with metformin ameliorates the imbalance and suppresses the release of IL-2 and TNF-α equally at both doses, i.e., 50 mg/kg and 80 mg/kg, in diabetic-treated groups. The suppression of these cytokines by metformin suggests that it can suppress immunomodulation by downregulating IL-2 and TNF-α. Metformin did not show any effect on immune parameters in healthy treated subjects, which highlights that metformin can suppress only the raised cytokines under pro-inflammatory conditions. Conclusively, it was identified that metformin is a good drug candidate that reduces pro-inflammatory cytokines IL-2, and TNF-α. Henceforth, further studies should be carried out to identify the appropriate dose for its immunomodulatory properties on various immune parameters and repurpose it as an immunomodulatory drug.
